# The Associations between Poor Antibiotic and Antimicrobial Resistance Knowledge and Inappropriate Antibiotic Use in the General Population Are Modified by Age

**DOI:** 10.3390/antibiotics11010047

**Published:** 2021-12-30

**Authors:** Huiling Guo, Zoe Jane-Lara Hildon, David Chien Boon Lye, Paulin Tay Straughan, Angela Chow

**Affiliations:** 1 Department of Clinical Epidemiology, Office of Clinical Epidemiology, Analytics, and Knowledge, Tan Tock Seng Hospital, Singapore 308433, Singapore; huiling_guo@ttsh.com.sg (H.G.); angela_chow@ttsh.com.sg (A.C.); 2 Saw Swee Hock School of Public Health, National University of Singapore, Singapore 117459, Singapore; 3 National University Health System, Singapore 119228, Singapore; 4 Infectious Disease Research and Training Office, National Centre for Infectious Diseases, Singapore 308443, Singapore; David_Lye@ncid.sg; 5 Yong Loo Lin School of Medicine, National University of Singapore, Singapore 117597, Singapore; 6 Department of Infectious Diseases, Tan Tock Seng Hospital, Singapore 308433, Singapore; 7 Lee Kong Chian School of Medicine, Nanyang Technological University, Singapore 308232, Singapore; 8 School of Social Sciences, Singapore Management University, Singapore 188065, Singapore; paulints@smu.edu.sg

**Keywords:** antimicrobial resistance, public knowledge, inappropriate antibiotic use, population-based survey, effect modification

## Abstract

Objectives: Understanding factors influencing inappropriate antibiotic use can guide the design of interventions to improve antibiotic practices and reduce antimicrobial resistance (AMR). Methods: A nationally representative cross-sectional survey (N = 2004) was conducted between November 2020 and January 2021. Knowledge of antibiotic use and AMR using the World Health Organization’s Multi-Country AMR Survey questionnaire, and antibiotic practices were examined. Multivariable logistic regression was performed to identify factors associated with inappropriate antibiotic use and examine effect measure modifications. Results: After adjusting for potential confounding, poor knowledge of antibiotic use was associated with a 3x increased odds of inappropriate antibiotic use in adults aged ≥50 years (aOR 3.11, 95% CI [2.24–4.32]), 5× increased odds in those aged 35–49 years (aOR 4.88, 95% CI [3.32–7.16]), and 7× increased odds in those aged 21–34 years (aOR 6.58, 95% CI [4.19–10.33]). While there was no statistically significant association in adults aged ≥50 years, poor knowledge of AMR increased the odds of inappropriate antibiotic use by 4 times in adults aged 35–49 years (aOR 3.73, 95% CI [1.53–9.11]) and 5 times in those aged 21–34 years (aOR 4.90, 95% CI [1.84–13.02]). Conclusions: Targeted educational interventions for specific age groups are needed in conjunction with empowering the public with knowledge of antibiotic use and AMR.

## 1. Introduction

Unlike influenza and coronavirus disease 2019 (COVID-19), which often result in explosive epidemics and pandemics, antimicrobial resistance (AMR) poses a silent but major threat to global health, with a projected annual mortality rate of 10 million deaths per year by 2050 [1]. Antibiotic misuse and overuse contribute significantly to the progression of AMR [2], and are often driven by the general population’s poor knowledge of appropriate antibiotic use and AMR [3,4,5].

A multi-country survey conducted by the World Health Organization (WHO) to assess public awareness of AMR amongst 12 countries in 2015 reported a lack of knowledge in both aspects [6]. More than half (64%) had the misconception that antibiotics are effective against viral infections; 32% believed that they should stop their prescribed course of antibiotics when they feel better, and 76% mistakenly thought that AMR is due to the human body, instead of bacteria, becoming resistant to antibiotics [6]. Several other studies reported similar observations due to poor antibiotic and AMR knowledge among the general public [7,8,9,10,11,12,13,14,15]. Population-based educational campaigns, however, have been found to have either mixed or no effect on improving knowledge and appropriate antibiotic practices, suggesting a need for more targeted approaches in tailoring education for specific sub-populations [16,17].

Population-based studies thus far have not assessed the knowledge gaps concerning antibiotics and AMR, stratified by socio-demographic factors. Purposive sampling from different sub-populations of the same local context is efficient but could lead to contradictory and non-generalisable findings due to selection bias [18,19]. Hence, in this study, we aimed to (1) assess the knowledge and practices related to antibiotic use and AMR of sub-populations in a nationally-representative population-based survey; (2) identify the unique determinants associated with inappropriate antibiotic use in sub-populations; and (3) identify potential areas for future targeted interventions tailored to sub-populations to improve antibiotic practices and reduce AMR.

## 2. Materials and Methods

### 2.1. Study Design and Sampling Frame

We conducted a nationally representative cross-sectional, community-based survey on a random sample of Singapore residents (citizens and permanent residents) aged 21 years and over between November 2020 and January 2021, using the sampling frame as described below:

Addresses with at least 1 Singapore resident were first stratified according to the ethnic group of the residents at the address. The number of addresses selected from each predominant ethnic group was proportionate to the distribution of the ethnic group in Singapore, with an additional oversampling of Malays and Indians to ensure representation from these minority groups in the final dataset. In addition, within each predominant ethnic group, the addresses were further stratified by their broad dwelling type. Within each detailed house type, addresses were randomly selected.

To avoid cluster bias, only one member of each household was invited to participate in the study. The person with the most recent birthday within each household was selected to avoid selection bias and to achieve a truly random sample of the population.

### 2.2. Survey Data Collection

The survey was self-administered and disseminated using a drop-off/pick-up method. Each survey package containing instructions on how to complete the hardcopy or online questionnaire was delivered in person via door-to-door visits. Any household uncontactable at the first visit was approached up to twice more at different times and on different days of the week before the household was replaced with a neighbouring household of the same housing type.

We developed the survey instrument through adaptation of questions and validated scales from various cross-sectional surveys reported in the literature [7,8,9,10,18,20,21,22,23,24]. Questions from the World Health Organization’s Antibiotic Resistance Multi-country Public Awareness Survey questionnaire were also incorporated to assess the knowledge, attitudes, and perceptions of antibiotic use and antibiotic resistance [6]. The survey instrument was developed in English and translated into 3 other languages: Malay, Mandarin, and Tamil.

The survey questionnaire focused on these main points: knowledge of antibiotics, antibiotic use and AMR, attitude towards antibiotics, antibiotic use and AMR, and self-reported antibiotic practices and experiences. Only the question items on knowledge and practice were used in this study. Knowledge questions on antibiotics, antibiotic use, and AMR were designed in the form of Yes/No/Don’t know or True/False/Don’t know, whilst practice questions were presented in a 5-point Likert scale: (1-Strongly disagree; 2-Disagree; 3-Neither agree nor disagree; 4-Agree; 5-Strongly agree). Respondents were determined to have good knowledge of antibiotic use and AMR after they had correctly answered all of the respective question items–7 items on antibiotic use and 8 on AMR (Supplementary Figures S1 and S2). Otherwise, they were deemed to have poor knowledge. Socio-demographic and medical background information was also collected: age, gender, ethnic group, residency status, education level, housing type, marital status, employment status, occupation, religious affiliation, and having a chronic illness and antibiotic experience.

### 2.3. Dependent Variable—Inappropriate Antibiotic Use

Six proxy statements on the inappropriate use of antibiotics were selected based on the US Centers for Disease Control and Prevention’s advisory on appropriate antibiotic use [25]: (1) “I normally keep antibiotic stocks at home in case of emergency”; (2) “if my family member is sick, I will usually give my antibiotics to them”; (3) “I will save leftover antibiotics for future use”; (4) “I normally stop taking antibiotics when I start feeling better”; (5) “I will see another doctor if my doctor does not give me antibiotics”; and (6) “I will take leftover antibiotics when I think I need them”. Respondents who agreed or strongly agreed to any of these 6 proxy statements were defined as inappropriate users of antibiotics.

### 2.4. Data Analysis

Proportions were tabulated for categorical variables, whereas means (standard deviations, SD) were calculated for continuous variables and responses on a 5-point Likert scale. Chi-squared test and t-test were used to compare differences between proportions and means, respectively.

Multivariable logistic regression was performed to determine the independent factors associated with inappropriate use of antibiotics. Covariates were selected through stepwise regression and included in the final regression model to adjust for potential confounding. Interactions between covariates were individually explored, and product terms were also included in the final model. Effect measure modification due to socio-demographic factors was further assessed. Statistical significance was defined as a *p*-value < 0.05. Statistical analyses were conducted in Stata version 14.0 (StataCorp LLC, College Station, TX, USA).

## 3. Results

### 3.1. Demographics of Participants

Out of 4791 households approached, 2004 (41.8%) responses were collected with demographics presented under Table 1.

The distribution of residency status (87% Singapore citizens; 13% permanent residents), gender (48% males; 52% females), ethnic group (72% Chinese, 15% Malay, 11% Indian, 3% Others); age (31% aged 21–34 years, 33% aged 35–49 years, 36% aged 50 years and above); housing type (6% 1- to 2-room flats, 20% 3-room flats, 35% 4-room flats, 20% 5-room and executive flats, 14% condominiums and other apartments, 5% landed properties); marital status (62% married, 30% never married, 8% others); and religious affiliation (82% had a religious affiliation, 18% had no religious affiliation) of our study population was representative of the Singapore population in 2020 [26]. Nearly two-thirds (65%) of them were higher educated, 70% were fully or partially employed, and 4% held healthcare-related professional positions. Furthermore, half (54%) of the respondents reported having a family member or friend working in the healthcare sector.

Of those who had a religious affiliation, one-third (33%) reported an influence of their religious beliefs on their health-seeking behaviour. Pertaining to health-related questions, 32% self-reported the presence of chronic illnesses, and 97% reported having taken antibiotics in their lifetime. Of those who had taken antibiotics, most had obtained them from a general practice clinic in Singapore (66%), 92% recalled receiving advice from a doctor, nurse, or pharmacist on how to take the antibiotics, and 81% reported completing them as prescribed.

### 3.2. Knowledge of Antibiotics and Antibiotic Use

The top 3 statements of knowledge answered correctly by respondents were “overuse of antibiotics can cause antibiotics to lose effectiveness in the long term” (77%), “antibiotics can treat bacterial infections” (72%), and “bacteria can be resistant to antibiotics” (68%) (Supplementary Figure S1). By contrast, more than half responded incorrectly that “infection by antibiotic-resistant bacteria can be easily cured” (61%), “antibiotics are the same as anti-inflammatory agents” (62%), and “antibiotics can treat viral infections” (65%). The statement revealing the greatest misconception was “humans can become resistant to antibiotics” (92%).

A total of 1188 Singapore residents (59%) answered correctly to all 3 knowledge statements on antibiotic use (Table 2).

The majority were aware that it is inappropriate to “use antibiotics that were given to a friend or family member, as long as they were used to treat the same illness” (89%), or “to buy the same antibiotics or request for them from a doctor, if they had helped you get better previously when you had the same symptoms” (68%), and 1703 (85%) respondents were aware that antibiotics should only be stopped when one has taken all of them as directed.

More than half responded correctly that bladder infection or urinary tract infection (64%) and skin or wound infection (57%) could be treated with antibiotics (Supplementary Table S1). However, less than half (39%) correctly responded that the common cold and flu do not warrant antibiotics. Interestingly, there was no difference in the proportion of correct responses concerning the use of antibiotics for the common cold or flu between respondents who had and had not heard of the annual national campaign “Fighting the flu virus is not my battle. Talk to your doctor for the treatment you need”, which was rolled out from 2018 and ran through 2020 [27], (49% compared to 50%, *p* = 0.876) (data not shown). Interestingly, 57% did not know that antibiotics could not be used to treat COVID-19, which is also a viral infectious disease.

### 3.3. Knowledge of AMR

The top 3 terms familiar to the respondents were “antibiotic resistance” (76%), “drug resistance” (69%), and “antibiotic-resistant bacteria” (64%) (Supplementary Table S2). While only 42% were aware of the official term used by WHO, i.e., “antimicrobial resistance”, “AMR” had been heard of by only 471 (24%) respondents. The predominant source of information was a doctor or nurse, followed by family or friends, mainstream media, and social media. The term “superbugs” had been heard of by 57% of our survey population, with one-quarter having heard of it from the mainstream media (26%), followed by social media (20%), or from a doctor or nurse (17%).

More than half of the respondents answered correctly to these statements: “if bacteria are resistant to antibiotics, it can be very difficult or impossible to treat the infections they cause” (67%), “antibiotic resistance is an issue that could affect my family or me” (61%), “many infections are becoming increasingly resistant to treatment by antibiotics” (59%), “antibiotic resistance is an issue in other countries but not here” (53%), and “antibiotic-resistant infections could make medical procedures like surgery, organ transplant, and cancer treatment much more dangerous” (51%) (Supplementary Figure S2). However, only one-third of the respondents were aware that “bacteria which are resistant to antibiotics can be spread from person to person” (34%), and the majority had the misconception that “antibiotic-resistant is only a problem for people who take antibiotics regularly” (63%), and “antibiotic resistance occurs when your body becomes resistant to antibiotics and they no longer work as well” (91%). In total, only 60 respondents (3%) answered correctly to all of the 8 knowledge statements on AMR.

### 3.4. Antibiotic Practices

Ninety percent of respondents reportedly professed to taking antibiotics according to instructions on the label, while 85% reported to have checked the expiry date of antibiotics before taking them (Supplementary Table S3). More than two-thirds of the respondents did not agree with statements concerning poor antibiotic practices such as stopping antibiotics when feeling better (70%), keeping antibiotic stocks at home in case of emergency (82%), stopping antibiotics after forgetting a dose (85%), saving leftover antibiotics for future use (88%), taking leftover antibiotics (89%), giving their antibiotics to sick family members (91%), and seeing another doctor if the current doctor did not prescribe antibiotics (94%). Overall, 61% (N = 1224) of respondents were observed to be inappropriate users of antibiotics.

To some extent, antibiotic practices regarding the common cold and flu were influenced by the COVID-19 pandemic (Supplementary Table S4). Respondents were more likely to take antibiotics to prevent their condition from getting worse (during the pandemic, mean 2.467 [SD 1.134]; pre-COVID-19 pandemic, mean 2.386 [SD 1.097]; *p* = 0.023) expected antibiotics from doctors for a cold or the flu (mean 2.570 [SD 1.140]; mean 2.484 [SD 1.118]; *p* = 0.016) during the COVID-19 pandemic than before.

### 3.5. Independent Factors Influencing Inappropriate Use of Antibiotics

Knowledge of antibiotic use, knowledge of AMR, gender, ethnic group, age, and education level were independent factors associated with the inappropriate use of antibiotics (Table 3).

Respondents with poor knowledge of antibiotic use (Model 1: aOR 4.30, 95% CI [3.46–5.33], *p* < 0.001) and on AMR (Model 1: aOR 3.07, 95% CI [1.72–5.49], *p* < 0.001) were three to four times as likely as those with good knowledge to be inappropriate users of antibiotics. There was an inverse dose-response relationship between inappropriate antibiotic use and age, with respondents aged 35–49 years (Model 1: aOR 1.42, 95% CI [1.10–1.81], *p* = 0.006) being 42% more likely and those aged 21–34 years (Model 1: aOR 2.16, 95% CI [1.63–2.88], *p* < 0.001) being twice as likely to be inappropriate users of antibiotics, compared to respondents aged 50 years and above.

Age was found to interact positively with poor knowledge of antibiotic use (aged 35–49 years: aOR 1.57, 95% CI [0.95–2.60], ns; aged 21–34 years: aOR 2.12, 95% CI [1.21–3.69], *p* = 0.008, with reference to aged ≥50 years) and poor knowledge of AMR (aged 35–49 years: aOR 5.25, 95% CI [1.05–26.30], *p* = 0.044; aged 21–34 years: aOR 6.88, 95% CI [1.30–36.34], *p* = 0.023, with reference to aged ≥50 years), and the interaction terms were included in Model 2. Males (Model 1: aOR 1.58, 95% CI [1.30–1.93], *p* < 0.001; Model 2: aOR 1.57, 95% CI [1.28–1.91], *p* < 0.001), non-Chinese (Model 1: aOR 1.30, 95% CI [1.02–1.65], *p* = 0.033; Model 2: aOR 1.27, 95% CI [1.00–1.61], *p* = 0.054), and lower educated respondents (Model 1: aOR 1.69, 95% CI [1.33–2.14], *p* < 0.001; Model 2: aOR 1.70, 95% CI [1.34–2.15], *p* < 0.001) were more likely to be inappropriate users of antibiotics.

With decreasing age, additive dose-response effects of poor knowledge of antibiotic use and AMR on inappropriate antibiotic use respectively were observed. After adjusting for knowledge of AMR, gender, ethnic group, highest education level, marital status, and reported religious influence on health-seeking behaviour, poor knowledge of antibiotic use increased the odds of being an inappropriate user of antibiotics by 3 times in respondents aged ≥ 50 years (aOR 3.11, 95% CI [2.24–4.32]), by 5 times in respondents aged 35–49 years (aOR 4.88, 95% CI [3.32–7.16]), and by 7 times in respondents aged 21–34 years (aOR 6.58, 95% CI [4.19–10.33]), respectively (Table 4).

Whilst there was no significant association between poor knowledge of AMR regarding inappropriate antibiotic use in respondents aged ≥ 50 years, poor knowledge of AMR increased the odds of being an inappropriate user of antibiotics by 4 times in respondents aged 35–49 years (aOR 3.73, 95% CI [1.53–9.11]), and 5 times in respondents aged 21–34 years (aOR 4.90, 95% CI [1.84–13.02]), respectively (Table 5).

### 3.6. Perceived Effectiveness of Promotional Methods for AMR Education

The top 3 promotional methods perceived to be effective by respondents regarding public education on AMR were posters or pamphlets in clinics or hospitals (66%), television and radio advertisements (63%), and annual AMR campaigns (61%) (Table 6).

Older adults aged ≥ 50 years were more likely to perceive traditional means such as posters or pamphlets in clinics or hospitals (*p* = 0.001), newspaper articles (*p* < 0.001), and magazine articles (*p* < 0.001) to be effective for public education on AMR, while younger adults aged 21–34 years were more likely to perceive contemporary methods such as YouTube (*p* < 0.001), movie advertisements (*p* = 0.001) and mobile applications (*p* = 0.001) as effective educational tools. Interestingly, respondents aged 35–49 years were more likely than their younger (21–34 years) and older (≥50 years) counterparts to perceive annual AMR campaigns to be effective (*p* = 0.013).

## 4. Discussion

Our study provided important insights concerning the knowledge and practices related to antibiotic use and AMR in the Singapore resident population. Poor knowledge of antibiotic use and AMR was evident, with a high proportion of respondents who thought that antibiotics are effective against viruses (65%), and did not know that antibiotic resistance does not occur when the body (instead of the bacteria) becomes resistant to antibiotics (91%). Compared to the aggregated findings from the WHO Antibiotic Resistance: Multi-country Public Awareness Survey [6], Singapore residents were more knowledgeable about the appropriate practices of antibiotic use. For instance, knowing that antibiotics should not be shared with others or requested from doctors when they are not clinically indicated, or that antibiotic prescriptions should be completed as directed. However, their knowledge of AMR lagged behind the respondents of the international study.

Awareness regarding medical conditions warranting antibiotics was also lacking, and therefore major knowledge gaps on antibiotic use and AMR need to be addressed. In particular, two consecutive years of consistent messaging through national AMR campaigns to correct the misuse of antibiotics for flu were carried out, but awareness that antibiotics cannot treat the common cold and flu remained low (39%), and the intervention showed no significant improvement in knowledge, supporting Price et al.’s argument that a “one-size-fits-all” public education was ineffective [16].

Another broader finding concerns the differences in antibiotic practices before and during the COVID-19 pandemic. With the majority of Singapore residents (61%) being inappropriate antibiotic users, there was also a slight increase of 3% in respondents reportedly using and expecting antibiotics to be prescribed inappropriately for cold or flu symptoms during the pandemic. This could be due to uncertainties in the clinical management of COVID-19, for which patients tended to present with acute respiratory symptoms. Notably, nearly half of the respondents did not know if antibiotics were required for COVID-19 treatment or not. More public education on COVID-19 is required to address the knowledge gaps.

Age played a pivotal role between knowledge and antibiotic practices. Poor knowledge of antibiotic use was associated with inappropriate antibiotic use in all age groups, with those aged 21–34 years having the strongest association. However, poor knowledge of AMR only had significant effects on those aged 35–49 years and 21–34 years, with increased odds of 4 and 5 times, respectively. As knowledge of AMR had no effect on the antibiotic use practices of individuals aged ≥50 years, addressing the knowledge deficit in AMR can be focused and tailored to adults below the age of 50. Whereas for older adults, as with their younger counterparts, education is needed to improve knowledge of the proper use of antibiotics. This finding is novel and not reported by previous studies.

Annual campaigns remained favourable for people across all ages (61%), but the roll-out of interventions could take on new forms leveraging technological advances and social networks. Traditional mass media, such as newspaper and magazine articles, were preferred by adults aged 50 years and above. Newer platforms such as YouTube were preferred by younger adults aged 21–34 years, as are advertisements during movies, mobile applications such as games, and online information portals. In particular, serious games or rules of “gamification” could be opportunistically used as they were shown to be successful in increasing knowledge of antibiotic use and AMR in both young children and adults [28,29,30]. Other socio-demographic factors identified were gender and education level, which further highlighted the need to conduct social network analysis, exploring each sub-population to identify key opinion leaders who could be crucial in influencing and driving appropriate use of antibiotics within the community.

Healthcare workers, in particular, doctors or nurses, could be tapped to provide public and patient education on appropriate antibiotic use and AMR. They have been reported as the top sources of information for terms related to AMR. Since the most preferred intervention was posters or pamphlets in clinics or hospitals (66%), they could be handed out during consultations as decision aids [31]. Previous studies have found that interactions with doctors, including medication counseling and shared decision-making for antibiotic treatment plans, were associated with better antibiotic practices by patients [32]. With nearly 80% of the study population having last received antibiotics from a primary care clinic (privately-funded GP clinic or publicly-funded polyclinic) in Singapore, primary care clinics can serve as critical avenues for opportunistic education on antibiotic use.

Primary healthcare providers are pivotal to providing education during a consultation, and shared decision-making concerning antibiotic uptake between patients and attending doctors should be encouraged, as recommended by the VALUE model [31]. In particular, the primary care setting is also seen as a key platform for educating the general public to the fact that the same antibiotics should not be bought or requested from a doctor simply because the medicine helped them get better previously when they had the same symptoms. In our survey, for instance, this was reportedly acceptable to nearly a third (32%) of study respondents.

This study has several strengths. Instead of convenience sampling, we adopted a robust methodology to avoid selection bias and cluster bias by proportionally stratifying the population by ethnic group (with oversampling of minority groups) and housing type to ensure representation, and randomly selecting one individual within each selected household. Despite a response rate of 41.8%, our study population was representative of the Singapore Census 2020, and therefore selection bias (if any) was likely to be minimal. As the population is heterogeneous in terms of education levels and language proficiencies, the survey booklets were provided in 3 other main languages (Malay, Mandarin, and Tamil) to avoid recruiting only English-literate residents and provide options to illiterate participants for survey administration by bilingual surveyors. Surveyors were all trained by the study team prior to data collection to avoid interviewer bias.

Furthermore, this is the first-ever nationally representative survey conducted in Singapore to assess residents’ knowledge and practices related to antibiotics and AMR. The adaptation of validated questions from the WHO Antibiotic Resistance: Multi-country Public Awareness Survey enabled international comparisons, and will allow future studies to assess the effectiveness of subsequent educational interventions. However, we cannot exclude the possibility of respondents underreporting inappropriate antibiotic practices due to social desirability bias, although it is likely to be minimal as the survey was anonymous and self-administered. Also, there could be unknown confounders which were not adjusted for in the final multivariable logistic regression model.

## 5. Conclusions

Knowledge deficits on antibiotic use and AMR are associated with the inappropriate use of antibiotics and how their effects are modified by age. Whilst education on antibiotic use should be considered for all adults, education on AMR could be tailored and targeted for adults aged below 50 years. Opportunistic education on antibiotic use at primary care clinics could be effective in improving antibiotic practices and reducing AMR.

## Figures and Tables

**Table 1:** Characteristics of 2004 Singapore residents surveyed between November 2020 and January 2021.

Demographics	Survey Respondents, %	Singapore Residents in Census 2020 ^a^, %
***Residency Status***		
Singapore Citizen	87	86
Permanent Resident	13	14
***Gender***		
Male	48	48
Female	52	52
***Ethnic Group***		
Chinese	72	76
Malay	15	13
Indian	11	8
Others	3	3
***Age Group***		
21–34 years old	31	26
35–49 years old	33	28
≥50 years old	36	46
***Highest Education Level***		
Lower Educated (Post-Secondary & below)	35	51
Higher Educated (Diploma & above)	65	49
***Housing Type* ^b^**		
HDB 1- and 2-Room Flats	6	6
HDB 3-Room Flats	20	18
HDB 4-Room Flats	35	32
HDB 5-Room and Executive Flats	20	23
Condominiums and Other Apartments	14	16
Landed Properties	5	5
***Marital Status***		
Married	62	63
Never Married	30	27
Others	8	10
***Has a Religious Affiliation***		
Yes	82	80
No	18	20
***Has Your Religion Ever Influenced Your Health-Seeking Behaviour?* ^c^**		
Yes	33	NA
No	67	NA
***Employment Status***		
Full-time Employment	62	NA
Part-time Employment	8	NA
Not Employed (Includes Unemployed and Retired)	30	NA
***Occupation***		
Non-Healthcare Related Roles	96	NA
Healthcare-Related Professional Roles	4	NA
***Family/Friend Working in Healthcare Sector***		
Yes	54	NA
No	46	NA
***Reported to Have At Least One Chronic Illness***		
Yes	32	NA
No	68	NA
***When was Antibiotics Last Taken?***		
In the Last Month	6	NA
In the Last 6 Months	16	NA
In the Last Year	17	NA
More than a Year Ago	36	NA
Cannot Remember	21	NA
Never	3	NA
***Source of Antibiotics (on Occasion Antibiotics Last Taken)* ^d^**		
GP Clinic in Singapore ^e^	66	NA
Polyclinic in Singapore ^f^	17	NA
Hospital in Singapore	10	NA
Overseas	1	NA
The Internet	0	NA
Friends or Family Members	0.2	NA
Antibiotics Saved up from Previous Time	1	NA
Cannot Remember	6	NA
***Ever Received Advice from a Doctor, Nurse or Pharmacist on How to Take the Antibiotics (on Occasion Antibiotics Last Taken)* ^d^**		
Yes	92	NA
No	3	NA
Cannot Remember	5	NA
***Ever Completed Prescribed Antibiotics Course (on Occasion Antibiotics Last Taken)* ^d^**		
Yes	81	NA
No	14	NA
Cannot Remember	6	NA

^a^ Includes population who are 20 years and above; ^b^ HDB flats refer to public housing provided by Singapore, and the number of rooms is a surrogate marker of a household’s socioeconomic status; ^c^ Of 1651 respondents who have a religious affiliation; ^d^ Of 1948 respondents who had ever taken antibiotics in their lifetime; ^e^ Privately-funded primary care clinic in Singapore; ^f^ Government-funded primary care clinic in Singapore. Abbreviations: HDB—Housing Development Board; GP—general practitioner.

**Table 2:** Proportion of correct responses provided by 2004 Singapore residents on statements related to antibiotic use.

Statement	Responses from 2004 Singapore Residents		
	Correct Response, %	Incorrect Response, %	Don’t Know, %
It is (NOT) Okay to Use Antibiotics That Were Given to a Friend or Family Member, As Long As They Were Used to Treat the Same Illness	89	6	6
It is (NOT) Okay to Buy the Same Antibiotics or Request for Them from a Doctor, If They had Helped You Get Better Previously When You Had the Same Symptoms	68	21	11
You Should Stop Antibiotics When You Have Taken All the Antibiotics As Directed Once You Have Begun Treatment	85	15	-

**Table 3:** Univariate and multivariable logistic regression analyses on factors influencing inappropriate use of antibiotics amongst 2004 Singapore residents surveyed between November 2020 and January 2021.

Variables	Appropriate Use of Antibiotics (N = 780)	Inappropriate Use of Antibiotics (N = 1224)	*p*-Value	Univariate Analysis (N = 2004)			Model 1 (N = 2004)			Model 2 (N = 2004)		
				Crude OR	95% CI	*p*-Value	Adjusted OR	95% CI	*p*-Value	Adjusted OR	95% CI	*p*-Value
**Knowledge of Antibiotic Use, N (%)**												
Poor Knowledge	151 (19)	665 (54)	**<0.001**	4.96	4.02–6.12	**<0.001**	4.30	3.46–5.33	**<0.001**	3.11	2.24–4.32	**<0.001**
**Knowledge of AMR, N (%)**												
Poor Knowledge	740 (95)	1204 (98)	**<0.001**	3.25	1.89–5.61	**<0.001**	3.07	1.72–5.49	**<0.001**	0.71	0.18–2.74	0.621
**Residency Status, N (%)**												
Singapore Citizen	673 (86)	1065 (87)	0.640	Ref	-	-	-	-	-	-	-	-
Permanent Resident	107 (14)	159 (13)		0.94	0.72–1.22	0.640	-	-	-	-	-	-
**Gender, N (%)**												
Male	319 (41)	635 (52)	**<0.001**	1.56	1.30–1.87	**<0.001**	1.58	1.30–1.93	**<0.001**	1.57	1.28–1.91	**<0.001**
**Ethnic Group, N (%)**												
Non-Chinese	166 (21)	400 (33)	**<0.001**	1.80	1.46–2.21	**<0.001**	1.30	1.02–1.65	**0.033**	1.27	1.00–1.61	0.054
**Age Group, N (%)**												
≥50 Years Old	321 (41)	410 (34)	**<0.001**	Ref	–	-	Ref	–	-	Ref	–	-
35–49 Years Old	276 (35)	382 (31)		1.08	0.88–1.34	0.460	1.42	1.10–1.81	**0.006**	0.24	0.05–1.19	0.081
21–34 Years Old	183 (23)	432 (35)		1.85	1.47–2.32	**<0.001**	2.16	1.63–2.88	**<0.001**	0.26	0.05–1.37	0.113
**Highest Education Level, N (%)**												
Higher educated (Diploma & above)	557 (71)	751 (61)	**<0.001**	Ref	-	-	Ref	-	-	Ref	-	-
Lower Educated (Post-Secondary & below)	223 (29)	473 (39)		1.57	1.30–1.91	**<0.001**	1.69	1.33–2.14	**<0.001**	1.70	1.34–2.15	**<0.001**
**Marital Status, N (%)**												
Never Married	253 (32)	499 (41)	**<0.001**	1.43	1.19–1.73	**<0.001**	1.19	0.96–1.49	0.117	1.18	0.95–1.48	0.136
**Employment Status, N (%)**												
Full-Time Employment	480 (62)	760 (62)	0.602	Ref	-	-	-	-	-	-	-	-
Part-Time Employment	59 (8)	105 (9)		1.12	0.80–1.58	0.499	-	-	-	-	-	-
Not Employed	241 (31)	359 (29)		0.94	0.77–1.15	0.548	-	-	-	-	-	-
**Occupation, N (%)**												
Non-Healthcare-Related Roles	510 (95)	841 (97)	**0.013**	1.99	1.15–3.46	**0.014**	-	-	-	-	-	-
**Family/Friend Working in Healthcare Sector, N (%)**												
No	338 (43)	590 (48)	**0.033**	1.22	1.02–1.46	**0.033**	-	-	-	-	-	-
**Reported to Have At Least One Chronic Illness, N (%)**												
No	503 (64)	853 (60)	**0.015**	1.27	1.05–1.53	**0.015**	-	-	-	-	-	-
**Has a Religious Affiliation, N (%)**												
Yes	645 (83)	1006 (82)	0.773	0.97	0.76–1.22	0.773	-	-	-	-	-	-
**Reported Religion Influence on Health-Seeking Behaviour, N (%)**												
Yes	189 (24)	350 (29)	**0.032**	1.25	1.02–1.54	**0.032**	1.16	0.91–1.46	0.226	1.14	0.90–1.44	0.277
Interaction between Knowledge of Antibiotic use and 35–49 Years Old	-	-	-	-	-	-	-	-	-	1.57	0.95–2.60	0.080
Interaction between Knowledge of Antibiotic use and 21–34 Years Old	-	-	-	-	-	-	-	-	-	2.12	1.21–3.69	**0.008**
Interaction between Knowledge of AMR and 35–49 Years Old	-	-	-	-	-	-	-	-	-	5.25	1.05–26.30	**0.044**
Interaction between Knowledge of AMR and 21–34 Years Old	-	-	-	-	-	-	-	-	-	6.88	1.30–36.34	**0.023**

Bolded values represent a statistical significance of *p*-value < 0.05.

**Table 4:** Association between inappropriate use of antibiotics and poor knowledge of antibiotic use, according to age group.

Inappropriate Use of Antibiotics	≥50 Years Old		35–49 Years Old			21–34 Years Old		
	OR	95% CI	OR	95% CI	*p*-Interaction ^a^	OR	95% CI	*p*-Interaction ^a^
**Unadjusted Analysis**								
Good Knowledge of Antibiotic Use	Ref	-	Ref	-	0.073	Ref	-	0.002
Poor Knowledge of Antibiotic use	3.35	2.43–4.63	5.27	3.62–7.66		8.03	5.16–12.48	
**Adjusted Analysis ^b^**								
Good Knowledge of Antibiotic Use	Ref	-	Ref	-	0.080	Ref	-	0.008
Poor Knowledge of Antibiotic Use	3.11	2.24–4.32	4.88	3.32–7.16		6.58	4.19–10.33	

^a^ Multiplicative scale; ^b^ Adjusted for knowledge of AMR, gender, ethnic group, highest education level, marital status and reported religion influence on health-seeking behaviour; Bolded values represent a statistical significance of *p*-value < 0.05.

**Table 5:** Association between inappropriate use of antibiotics and poor knowledge of AMR, according to age group.

Inappropriate Use of Antibiotics	≥50 Years Old		35–49 Years Old			21–34 Years Old		
	OR	95% CI	OR	95% CI	*p*-Interaction ^a^	OR	95% CI	*p*-Interaction ^a^
**Unadjusted Analysis**								
Good Knowledge of AMR	Ref	-	Ref	-	0.111	Ref	-	**0.034**
Poor Knowledge of AMR	1.02	0.27–3.84	3.65	1.58–8.42		5.82	2.35–14.39	
**Adjusted Analysis ^b^**								
Good Knowledge of AMR	Ref	-	Ref	-	**0.044**	Ref	-	**0.023**
Poor Knowledge of AMR	0.71	0.18–2.74	3.73	1.53–9.11		4.90	1.84–13.02	

^a^ Multiplicative scale; ^b^ Adjusted for knowledge of antibiotic use, gender, ethnic group, highest education level, marital status, and reported religious influence on health-seeking behaviour; Bolded values represent a statistical significance of *p*-value < 0.05.

**Table 6:** Proportion of 2004 Singapore residents on their perceived effectiveness for a list of promotional methods to educate on AMR, stratified by age group.

Promotional Method	Total, %	21–34 Years Old, %	35–49 Years Old, %	≥50 Years Old, %	*p*-Value
Posters or Pamphlets in Clinics or Hospitals	66	60	68	69	**0.001**
Television and Radio Advertisements	63	61	63	63	0.734
Annual Campaigns (e.g., World Antibiotics Awareness Week)	61	57	65	62	**0.013**
Newspaper Articles	56	47	59	62	**<0.001**
Social Media (e.g., Facebook, Instagram, WhatsApp)	51	59	56	41	**<0.001**
Posters at Bus Stops	48	50	50	45	0.079
YouTube	47	54	48	40	**<0.001**
Magazine Articles	43	32	46	49	**<0.001**
Movie Advertisements	41	45	42	35	**0.001**
Mobile Applications (e.g., Games, Information Portals)	37	40	39	31	**0.001**

Bolded values represent a statistical significance of *p*-value < 0.05.

## Data Availability

The data presented in this study are contained within this article and the Supplementary Materials.

## Supplementary Materials

The following supporting information can be downloaded at: https://www.mdpi.com/article/10.3390/antibiotics11010047/s1, Figure S1: Proportion of correct responses provided by 2004 Singapore residents on statements related to knowledge of antibiotics, Figure S2: Proportion of correct responses provided by 2004 Singapore residents on statements related to AMR; Table S1: Proportion of correct responses provided by 2004 Singapore residents on which medical condition warrants antibiotics, Table S2: Proportion of 2004 Singapore residents who had ever heard of terms related to AMR, Table S3: Proportion and mean score of self-reported antibiotic practices of 2004 Singapore residents on a 5-point Likert scale, Table S4: Proportion and mean score of self-reported antibiotic practices of 2004 Singapore residents for common cold and flu, before and during COVID-19 pandemic, on a 5-point Likert scale.

## References

[1] Review on Antimicrobial Resistance (2014). Antimicrobial Resistance: Tackling a Crisis for the Health and Wealth of Nations. https://amr-review.org/sites/default/files/AMR%20Review%20Paper%20-%20Tackling%20a%20crisis%20for%20the%20health%20and%20wealth%20of%20nations_1.pdf.

[2] World Health Organization (2020). Antibiotic resistance. https://www.who.int/news-room/fact-sheets/detail/antibiotic-resistance.

[3] Choo S.J., Chang C.T., Lee J.C.Y., Munisamy V., Tan C.K., Raj J.D., Taib R.I.M., Thong K.S., Shafie A.A. (2018). A cross-sectional study on public belief, knowledge and practice towards antibiotic use in the state of Perak, Malaysia. J. Infect. Dev. Ctries..

[4] Lim J.M., Chhoun P., Tuot S., Om C., Krang S., Ly S., Hsu L.Y., Yi S., Tam C.C. (2021). Public knowledge, attitudes and practices surrounding antibiotic use and resistance in Cambodia. JAC Antimicrob. Resist..

[5] Gillani A.H., Chang J., Aslam F., Saeed A., Shukar S., Khanum F., Jairoun A., Nicholson A., Ibrahim M.I.M., Fang Y. (2021). Public knowledge, attitude, and practice regarding antibiotics use in Punjab, Pakistan: A cross-sectional study. Expert Rev. Anti-Infect. Ther..

[6] World Health Organization (2015). Antibiotic Resistance: Multi-Country Public Awareness Survey.

[7] Oh A.L., Hassali M.A., Al-Haddad M.S., Sulaiman S.A.S., Shafie A.A., Awaisu A. (2011). Public knowledge and attitudes towards antibiotic usage: A cross-sectional study among the general public in the state of Penang, Malaysia. J. Infect. Dev. Ctries..

[8] Awad A.I., Aboud E.A. (2015). Knowledge, attitude and practice towards antibiotic use among the public in Kuwait. PLoS ONE.

[9] Gualano M.R., Gili R., Scaioli G., Bert F., Siliquini R. (2015). General population’s knowledge and attitudes about antibiotics: A systematic review and meta-analysis. Pharmacoepidemiol. Drug Saf..

[10] Yusef D., Babaa A.I., Bashaireh A.Z., Al-Bawayeh H.H., Al-Rijjal K., Nedal M., Kailani S. (2018). Knowledge, practices & attitude towards antibiotics use and bacterial resistance in Jordan: A cross-sectional study. Infect. Dis. Health.

[11] Almohammed R.A., Bird E.L. (2019). Public knowledge and behaviours relating to antibiotic use in Gulf Cooperation Council countries: A systematic review. J. Infect. Public Health.

[12] Michaelidou M., Karageorgos S.A., Tsioutis C. (2020). Antibiotic use and antibiotic resistance: Public awareness survey in the Republic of Cyprus. Antibiotics.

[13] Chukwu E.E., Oladele D.A., Awoderu O.B., Afocha E.E., Lawal R.G., Abdus-Salam I., Ogunsola F.T., Audu R.A. (2020). A national survey of public awareness of antimicrobial resistance in Nigeria. Antimicrob. Resist. Infect. Control..

[14] Kosiyaporn H., Chanvatik S., Issaramalai T., Kaewkhankhaeng W., Kulthanmanusorn A., Saengruang N., Witthayapipopsakul W., Viriyathorn S., Kirivan S., Kunpeuk W. (2020). Surveys of knowledge and awareness of antibiotic use and antimicrobial resistance in general population: A systematic review. PLoS ONE.

[15] Zaidi S.F., Baroom M.W., Hanbashi A.I., Alkhaibari A.A., Yahya A.O., Alsalmi M., Alotaibi R., Nagro A., Khan M.A., Alshanberi A.M. (2021). Cross-sectional survey among general population regarding knowledge and attitude toward antibiotic usage in Western Saudi Arabia. Pharmacy.

[16] Price L., Gozdzielewska L., Young M., Smith F., MacDonald J., McParland J., Williams L., Langdridge D., Davis M., Flowers P. (2018). Effectiveness of interventions to improve the public’s antimicrobial resistance awareness and behaviours associated with prudent use of antimicrobials: A systematic review. J. Antimicrob. Chemother..

[17] Lim J.M., Singh S.R., Duong M.C., Legido-Quigley H., Hsu L.Y., Tam C.C. (2020). Impact of national interventions to promote responsible antibiotic use: A systematic review. J. Antimicrob. Chemother..

[18] Pan D.S.T., Huang J.H., Lee M.H.M., Yu Y., Chen M.I., Goh E.H., Jiang L., Chong J.W.C., Leo Y.S., Lee T.H. (2016). Knowledge, attitudes and practices towards antibiotic use in upper respiratory tract infections among patients seeking primary health care in Singapore. BMC Fam. Pract..

[19] Tam C., Lim J. (2019). Commentary: Our Misuse, Overuse of Antibiotics Comes with a Huge Cost.

[20] André M., Vernby A., Berg J., Lundborg C.S. (2010). A survey of public knowledge and awareness related to antibiotic use and resistance in Sweden. J. Antimicrob. Chemother..

[21] Wun Y.T., Lam T.P., Lam K.F., Sun K.S. (2012). Antibiotic use: Do parents act differently for their children?. Int. J. Clin. Pract..

[22] Lim K.K., Teh C.C. (2012). A cross sectional study of public knowledge and attitude towards antibiotics in Putrajaya, Malaysia. South. Med. Rev..

[23] Zhuo A., Labbate M., Norris J.M., Gilbert G.L., Ward M.P., Bajorek B.V., Degeling C., Rowbotham S.J., Dawson A., Nguyen K.-A. (2018). Opportunities and challenges to improving antibiotic prescribing practices through a One Health approach: Results of a comparative survey of doctors, dentists and veterinarians in Australia. BMJ Open.

[24] Centre for Health Protection Department of Health (2017). General Public’s Knowledge, Attitude and Practice Survey on Antimicrobial Resistance 2016/17.

[25] Centers for Disease Control and Prevention (2021). Antibiotic Do’s & Don’ts: Take antibiotics exactly as prescribed if you need them. https://cdc.gov/antibiotic-use/do-and-dont.html.

[26] Department of Statistics Singapore (2021). Singapore Census of Population 2020.

[27] Health Promotion Board (2021). Programmes: Antibiotics do not treat flu. https://healthhub.sg/programmes/146/use-antibiotics-right.

[28] Eley C.V., Young V.L., Hayes C.V., Verlander N.Q., McNulty C.A.M. (2019). Young people’s knowledge of antibiotics and vaccinations and increasing this knowledge through gaiming: Mixed-methods study using e-Bug. JMIR Serious Games.

[29] Tsopra R., Courtine M., Sedki K., Eap D., Cabal M., Cohen S., Bouchaud O., Mechaï F., Lamy J.-B. (2020). AntibioGame^®^: A serious game for teaching medical students about antibiotic use. Int. J. Med. Inform..

[30] Aboalshamat K., Khayat A., Halwani R., Bitan A., Alansari R. (2020). The effects of gamification on antimicrobial resistance knowledge and its relationship to dentistry in Saudi Arabia: A randomized controlled trial. BMC Public Health.

[31] Guo H., Hildon Z.J., Loh V.W.K., Sundram M., Ibrahim M.A.B., Tang W.E., Chow A. (2021). Exploring antibiotic prescribing in public and private primary care settings in Singapore: A qualitative analysis informing theory and evidence-based planning for value-driven intervention design. BMC Fam. Pract..

[32] Zanichelli V., Tebano G., Gyssens I.C., Vlahović-Palčevski V., Monnier A.A., Benic M.S., Harbarth S., Hulscher M., Pulcini C., Huttner B.D. (2019). Patient-related determinants of antibiotic use: A systematic review. Clin. Microbiol. Infect..

