# Supplementary material for: The Associations between Poor Antibiotic and Antimicrobial Resistance Knowledge and Inappropriate Antibiotic Use in the General Population Are Modified by Age
Source: Antibiotics (Basel). 2021 Dec 30;11(1):47. doi: 10.3390/antibiotics11010047 (PMC8773329; doi:10.3390/antibiotics11010047)
Supplement: Supplementary file 1 [file antibiotics-11-00047-s001.zip › antibiotics-1495393-supplementary.pdf]

Figure S1. Proportion of correct responses provided by 2004 Singapore residents on statements related to knowledge of antibiotics.

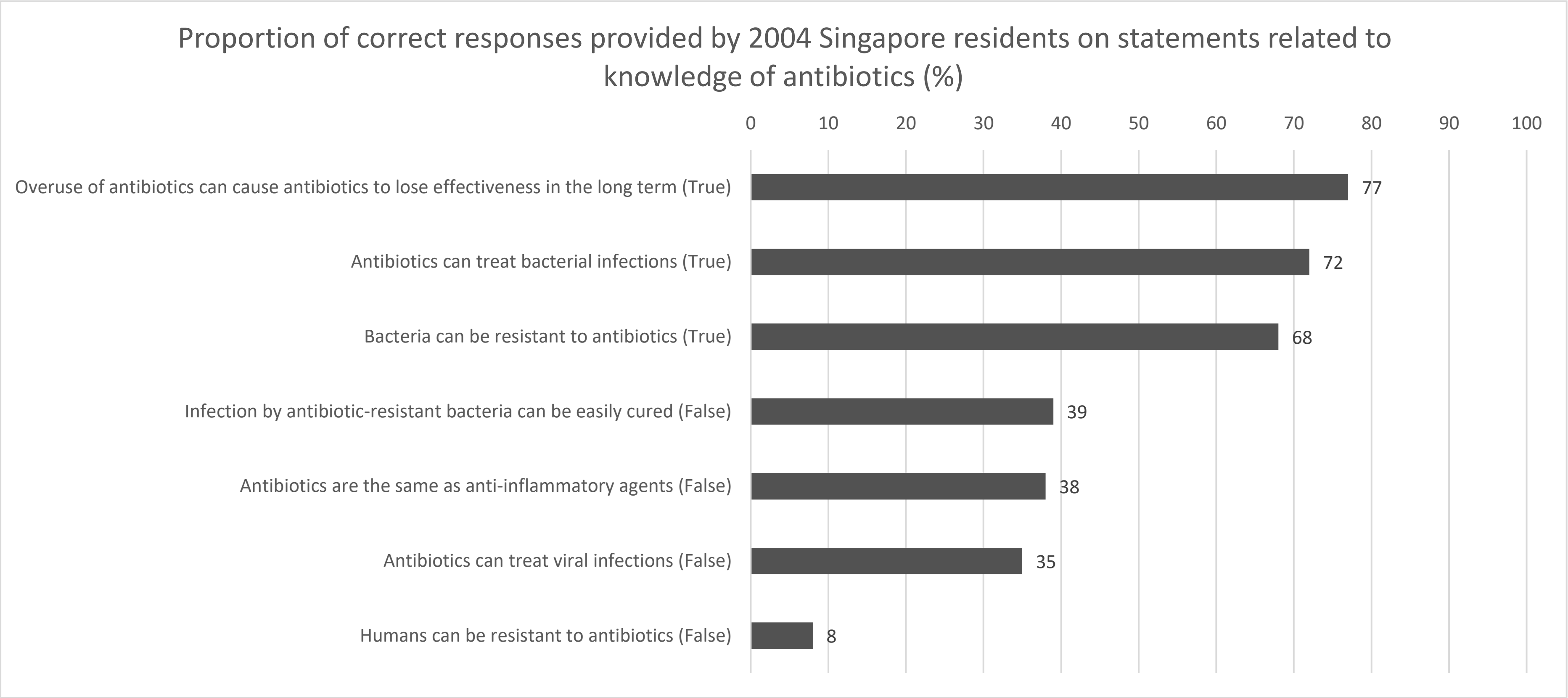

Table S1. Proportion of correct responses provided by 2004 Singapore residents on which medical condition warrants antibiotics.

Medical condition	Responses from 2004 Singapore residents		
	Correct response, %	Incorrect response, %	Don't know, %
Bladder infection or urinary tract infection	64	10	26
Skin or wound infection	57	20	23
Common cold or flu	39	47	13
COVID-19	43	12	45

Supplementary Table S2: Proportion of 2004 Singapore residents who had ever heard of terms related to AMR

Term	Responses from 2004 Singapore residents		
	**Ever heard of this term, %**	**Top 3 sources of information, %**	
Antibiotic resistance	76	Doctor or nurse:<br>Media (TV, radio, etc.):<br>Family or friend:	52<br>29<br>27
Drug resistance	69	Doctor or nurse:<br>Media (TV, radio, etc):<br>Social media:	31<br>23<br>19
Antibiotic-resistant bacteria	64	Doctor or nurse:<br>Media (TV, radio, etc):<br>Social media:	26<br>21<br>18
Superbugs	57	Media (TV, radio, etc):<br>Social media:<br>Doctor or nurse:	26<br>20<br>17
Antimicrobial resistance	42	Doctor or nurse:<br>Media (TV, radio, etc.):<br>Social media:	32<br>30<br>26
AMR	24	Doctor or nurse:<br>Social media:<br>Media (TV, radio, etc.):	32<br>15<br>12

Supplementary Figure S2: Proportion of correct responses provided by 2004 Singapore residents on statements related to AMR

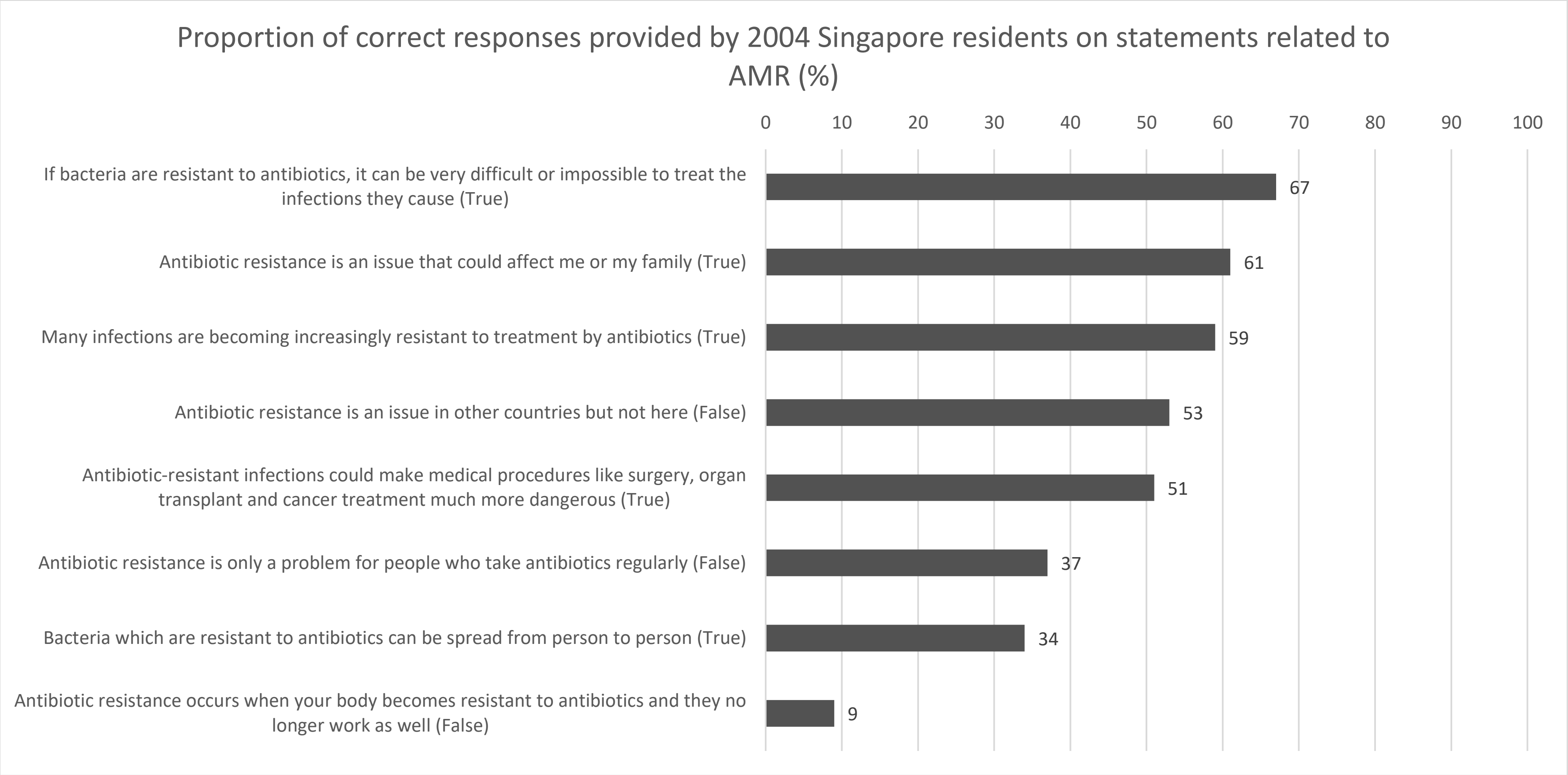

Supplementary Table S3: Proportion and mean score of self-reported antibiotic practices of 2004 Singapore residents on a 5-point Likert scale

**Statement**	**Strongly agree/Agree, %**	**Neither agree nor disagree, %**	**Strongly disagree/ Disagree, %**	**Mean (SD)**
I will take antibiotics according to the instructions on the label	90	5	5	4.294 (0.894)
I will normally look at the expiry date of antibiotics before taking them	85	7	8	4.216 (0.990)
I normally stop taking antibiotics when I start feeling better	30	9	61	2.512 (1.314)
I normally keep antibiotic stocks at home in case of emergency	18	15	68	2.273 (1.133)
I might stop taking the antibiotic after I forget a dose	15	16	69	2.277 (1.016)
I will save leftover antibiotics for future use	12	9	79	1.873 (1.087)
I will take leftover antibiotics when I think I need them	11	8	81	1.891 (1.017)
If my family member is sick, I will usually give my antibiotics to them	9	11	81	1.837 (1.005)
I will see another doctor if my doctor does not give me antibiotics	6	13	81	1.919 (0.871)

Supplementary Table S4: Proportion and mean score of self-reported antibiotic practices of 2004 Singapore residents for common cold and flu, before and during COVID-19 pandemic, on a 5-point Likert scale

**Statement**	**Strongly agree/Agree, N (%)**	**Neither agree nor disagree, N (%)**	**Strongly disagree/ Disagree, N (%)**	**Mean (SD)**	***P*-value**
Before the COVID-19 pandemic, when I get a cold/flu, I will take antibiotics to help me get better more quickly	16	18	66	2.284 (1.085)	0.229
During the COVID-19 pandemic, when I get a cold/flu, I will take antibiotics to help me get better more quickly	16	22	62	2.326 (1.092)	
Before the COVID-19 pandemic, when I get a cold/flu, I will take antibiotics to prevent my condition from getting worse	19	21	61	2.386 (1.097)	**0.023**
During the COVID-19 pandemic, when I get a cold/flu, I will take antibiotics to prevent my condition from getting worse	21	22	57	2.467 (1.134)	
Before the COVID-19 pandemic, I expect antibiotics to be prescribed by my doctor if I suffer from common cold/flu symptoms	21	23	56	2.484 (1.118)	**0.016**
During the COVID-19 pandemic, I expect antibiotics to be prescribed by my doctor if I suffer from common cold/flu symptoms	24	24	52	2.570 (1.140)	
